# Physical Demand Profiles of Hatha Yoga Postures Performed by Older Adults

**DOI:** 10.1155/2013/165763

**Published:** 2013-10-03

**Authors:** George J. Salem, Sean S.-Y. Yu, Man-Ying Wang, Sachithra Samarawickrame, Rami Hashish, Stanley P. Azen, Gail A. Greendale

**Affiliations:** ^1^Division of Biokinesiology and Physical Therapy, University of Southern California (USC), 1540 E. Alcazar Street, Los Angeles, CA 90033, USA; ^2^Department of Preventive Medicine, Keck School of Medicine, University of Southern California, USA; ^3^Division of Geriatrics, Geffen School of Medicine at the University of California, Los Angeles (UCLA), 924 Westwood Boulevard, Suite 200, Los Angeles, CA 90024, USA

## Abstract

Understanding the physical demands placed upon the musculoskeletal system by individual postures may allow experienced instructors and therapists to develop safe and effective yoga programs which reduce undesirable side effects. Thus, we used biomechanical methods to quantify the lower extremity joint angles, joint moments of force, and muscle activities of 21 Hatha yoga postures, commonly used in senior yoga programs. Twenty older adults, 70.7 years ± 3.8 years, participated in a 32-wk yoga class (2 d/wk) where they learned introductory and intermediate postures (asanas). They then performed the asanas in a motion analysis laboratory. Kinematic, kinetic, and electromyographic data was collected over three seconds while the participants held the poses statically. Profiles illustrating the postures and including the biomechanical data were then generated for each asana. Our findings demonstrated that Hatha yoga postures engendered a range of appreciable joint angles, JMOFs, and muscle activities about the ankle, knee, and hip, and that demands associated with some postures and posture modifications were not always intuitive. They also demonstrated that all of the postures elicited appreciable rectus abdominis activity, which was up to 70% of that induced during walking.

## 1. Introduction

Yoga has traditionally been viewed as a relatively safe form of exercise, capable of increasing strength, flexibility, endurance, balance, and functional capacity of people in good health and those with musculoskeletal disorders [1–5]. Supporting these postulates, the US Department of Health and Human Services and the National Recreation and Park Association have recommended yoga as a form of “total-solution” exercise for older adults [6]. Despite these dramatic claims of improved function across a range of physiological and psychosocial systems, little is understood regarding the physical demands, program efficacy, and overall safety of yoga programs for older adults. In general, older adults have less strength, joint flexibility, and balance, compared to younger adults. Moreover, they have a greater prevalence of osteoarthritis and neurological syndromes (e.g., sciatica and spinal-canal stenosis)—putting them at higher risk of developing exercise-related musculoskeletal and neurological complications. Understanding the physical demands placed upon the musculoskeletal system by individual postures may allow experienced instructors and therapists, whom have specialized in training with senior populations, to develop safe and effective yoga programs which reduce these undesirable side effects.

This, in essence, was the primary goal of the Yoga Empowers Seniors Study (YESS)—to quantify the physical demands associated with the performance of individual postures (asanas) and posture modifications frequently used in senior yoga programs [7–9]. The purpose of the present paper from the YESS project is to describe asana-specific lower-extremity (LE) demands placed on the practitioners by the 9 introductory and the 12 intermediate asanas used in the YESS project.

The physical demands of asanas are quantified biomechanically using 3D motion analysis, force platforms, and electromyography (EMG). While performing an asana, gravitational forces tend to rotate our arms and legs and pull our body towards the earth. In order to “hold” a posture and prevent our limbs from rotating, we must use our muscles and ligaments to resist these gravitational effects. We can quantify these muscular and ligamentous “efforts” by calculating the joint moments of force (JMOFs) produced about the joints of the body during the performance of an asana. Because the JMOFs are related to the torque that a muscle must develop while holding a posture, they provide insight into the specific muscle groups that are used during asana performance. Knowledge of the muscles that are working informs the beneficial adaptations (e.g., increased strength and endurance) that we would expect to occur. JMOFs can also provide a window to potential injury, because excessively-high JMOFs can create detrimental loading of articular, ligamentous, and capsular structures, essentially overloading the musculoskeletal system. Therefore, JMOFs can also be used to select postures that avoid usage of injured or overtaxed muscles and tissues. We also recorded the muscle activity of selected muscle groups using the electromyographic (EMG) analysis. Surface recording of the electrical activity of major muscles provides a complementary window on the physical demands of each posture. Aggregating the biomechanical profiles (JMOF, EMG, and maximum joint angles) of each posture will allow the design of the asana series that are well-balanced—targeting all of the functionally important muscle groups without repeatedly overloading the same musculoskeletal and articular tissues. Knowledge of the physical demands of each posture can also be used by experienced teachers and therapists, whom have specialized in training with senior populations, to select optimal asanas for their students, for example, focusing on postures that would strengthen weak muscle groups and/or unload injured and healing structures. In addition, especially for senior practitioners, a well-designed series will avoid the excessive range of motion in joints that are particularly susceptible to injury such as the knees and hips. 

## 2. Methods

### 2.1. Study Design

The design of YESS has been previously detailed [9]. In brief, YESS was an intervention development study designed to quantify the physical demands of selected Hatha yoga postures and modifications in ambulatory senior men and women. Participants attended 1-hour Yoga classes, 2 days per week, for 32 weeks. For the first 16 weeks, they were taught an introductory series, and for the second 16 weeks they were advanced to an intermediate series. The classes were led by a yoga instructor (YT500 certification) with considerable (over 10 years) experience in teaching seniors including teaching in prior research projects conducted by our group. A research associate assisted at the classes. The RA had collegiate gymnastic athletic training experience (2 years); further, she was specifically mentored in how to assist in yoga classes by both a PI (GAG) and the yoga instructor. Biomechanical data, including maximum joint angles, JMOF, and muscle activation levels, were collected after 16 (introductory postures) and 32 weeks (intermediate postures) of yoga practice. Participant recruitment and the yoga classes were conducted at the University of California Los Angeles (UCLA) and TruYoga studio (Santa Monica, CA), respectively. Biomechanical data was collected at the Musculoskeletal Biomechanics Research Laboratory (MBRL) at the University of Southern California (USC). Both the USC and UCLA Institutional Review Boards approved the study protocol, and all participants provided informed, written consent.

### 2.2. Inclusion/Exclusion Criteria

Inclusion/exclusion criteria were selected in order to maximize safe participation while in the yoga classes and during the testing sessions. Community dwelling men and women volunteers, aged 65 years or older, who were not high-level exercisers or frequent long walkers, and were yoga novices, were eligible for the study. High level exercisers were defined as people who participated in active sports (e.g., aerobics, jogging, and tennis) or higher-intensity exercises (>6 MET). Frequent long walkers were defined as those who walked more than a mile without resting, at least 3 times per week. The following safety exclusions were adopted in order to decrease potential cardiovascular, musculoskeletal, and neurological risks to the participants: active angina; uncontrolled hypertension (SBP greater than 160 or DBP greater than 90); high resting pulse or respiratory rate (HR > 90 or RR > 24 after 5 minutes seated); unstable asthma or exacerbated COPD; cervical spine instability or other significant neck injury; rheumatoid arthritis; unstable ankle, knee, hip, shoulder, elbow, or wrist joints; hemiparesis or paraparesis; movement disorders (e.g., Parkinson's disease), peripheral neuropathies, stroke with residual deficits, and severe vision or hearing problems; walker or wheelchair use; insufficient hearing to permit safety in a yoga group setting; inability to attend in-person classes; not having a checkup by regular provider within 12 months (if not taking any prescription medications) or in the past 6 months (if any regular medicines taken); could not pass specific movement safety tests. The qualifying movements were the ability to (a) get up from the floor to standing; (b) go from standing to the floor; (c) lift both arms to shoulder level without losing balance; (d) stand with feet side-by-side for 30 seconds; and (e) stand with feet hip-width apart for 60 seconds. These were assessed by the study PI and/or an experienced research associate. The following *feasibility/adherence* exclusions were also utilized: (1) the inability to understand their commitment to the project (laboratory visits and regular program participation) and (2) the cognitive limitations significant enough to preclude informed consent or to raise concerns about participation safety. 

### 2.3. Sample Size and Recruitment

A target sample size of 20 was determined *a-priori* using a power-analysis of pilot data comparing JMOFs across asanas in a sample of 3 older adults. Recruitment was initiated on January 7, 2009 and ended on March 5, 2010. Potential participants (*n* = 114) were contacted and initially screened over the phone. Screening was conducted by the Project Director (PD) (with 10 years of experience) and the Research Associate (RA) (2 years of experience). The latter was closely supervised by the former. 79 participants passed the phone screening exam and 26 were elected not to participate. Of these 79, 46 passed the in-person screening and were allocated to Group 1 (*n* = 15), Group 2 (*n* = 15), or waitlisted. Participants were again screened at the baseline to insure no conditions had arisen that would exclude the participants, and that no previously undetected conditions were present. In Group 1, 12 participants passed the baseline exam, and in Group 2, 15 participants passed the baseline exam. Thus, 27 participants were enrolled and had baseline measures taken. 

### 2.4. Retention

Within Group 1, 4 participants left the study for the following reasons: (1) time commitment was deemed too great (*n* = 2); (2) one subject failed to attend 3 of the 4 initial yoga classes due to travel; (3) one subject informed the instructor during the second class that a previous spine surgeon had instructed her to “not rotate her neck.” She had not disclosed this information previously and follow-up contact with the physician resulted in her being removed from the study. In Group 2, 3 participants left the study: (1) 1 subject injured her knee the week prior to the beginning of the study and could not attend the initial yoga classes; (2) 1 subject had a return of previously diagnosed bilateral, posterior thigh pain following the baseline testing and then found the initial yoga classes “difficult.” After receiving epidural injection without much improvement, physician and PI concluded yoga would not be advisable at the present time; (3) 1 subject had low back pain that did not resolve with rest; thus, PI decided pain could become worse with yoga. Thus, a total of 20 participants, 8 in Group 1 and 12 in Group 2, were able to complete the yoga program and had biomechanics measures taken at 16 and 32 weeks. The average age of the 14 women and 6 men was 70.7 years ± 3.8 yr.

### 2.5. Yoga Program

The study employed Hatha yoga, which incorporates *asanas* and *pranayama* (breathing). The program incorporated a standard set of opening and closing sequences and 2 ordered progressive middle sequences, termed series I (first 16 weeks) and series II (second 16 weeks). Predicated on our own experiences, as well as through reviewing videos, books, and websites aimed at seniors [10–12], each series included postures and pose modifications that (1) were commonly used in senior yoga programs; (2) we believed it could be performed safely by seniors in a group environment; and (3) provided a balanced and comprehensive fitness program that targeted muscle groups thought to be integral to conducting activities of daily living. The postures that were investigated are listed below. The introductory postures were advanced by removing or modifying the use of props, or moving from a bilaterally-supported posture to a unilaterally-supported posture. For example, during performance of the *introductory* side stretch asana, the participants supported their stance by placing their hands approximately chest high against a wall. For the *intermediate* side stretch posture, the participants lowered their support height by placing their hands on the backrest of a chair. The tree posture was advanced by having the participants stand on a single limb without use of a wall in the *intermediate* version. During the *introductory* tree posture, the participants lightly touched a wall and had their nonsupporting limb touching the floor. During the *introductory* warrior II asana, the participants supported themselves by lightly touching a chair. Contrastingly, the *intermediate* warrior II posture was performed without the use of a chair. The introductory postures are

 Chair (Utkatasana) with wall (Figure 2)
 Tree (Vrksasana) bilateral and wall (Figure 3)
 Downward dog (Adho Mukha Svanasana) with wall (Figure 4)
 Warrior I (Virabhadrasana I) with chair (front) (Figure 5)
 Warrior I (Virabhadrasana I) with chair (back) (Figure 6)
 Warrior II (Virabhadrasana II) with chair (front) (Figure 7)
 Warrior II (Virabhadrasana II) with chair (back) (Figure 8)
 Side stretch (Parsvottanasana) with wall (front) (Figure 9)
 Side stretch (Parsvottanasana) with wall (back) (Figure 10)

Intermediate Postures

 Chair (Utkatasana) (Figure 11)
 Tree (Vrksasana) unilateral and wall (Figure 12)
 Tree (Vrksasana) unilateral (Figure 13)
 Warrior II (Virabhadrasana II) (front) (Figure 14)
 Warrior II (Virabhadrasana II) (back) (Figure 15)
 Side stretch (Parsvottanasana) with chair (front) (Figure 16)
 Side stretch (Parsvottanasana) with chair (back) (Figure 17)
 One-leg balance (Utthita Hasta Padangusthasana) with blocks (Figure 18)
 One-leg balance (Utthita Hasta Padangusthasana) with chair (Figure 19)
 One-leg balance (Utthita Hasta Padangusthasana) unilateral (Figure 20)
 Crescent (Ashta Chandrasana) (front) (Figure 21)
 Crescent (Ashta Chandrasana) (back) (Figure 22)

### 2.6. Biomechanics

The biomechanical outcome variables examined included (1) average maximum joint angles, (2) average peak net JMOFs, and (3) average peak EMG activity engendered during the performance of the individual yoga postures. Biomechanical analysis was performed at the USC Musculoskeletal Biomechanics Research Laboratory using standard techniques [8, 13]. Whole body kinematic data were collected using an eleven-camera motion capture system at 60 Hz (Qualisys Tracking System with Oqus 5 cameras; Qualisys, Gothenburg, Sweden). Reflective markers were placed on a head band and over the following anatomical landmarks of the lower and upper extremities bilaterally: first and fifth metatarsal heads, malleoli, femoral epicondyles, greater trochanters, acromions, greater tubercles, humeral epicondyles, radial and ulnar styloid processes, and third metacarpal heads. Markers were also attached to the spinous process of the 7th cervical vertebra (C7), jugular notch, L5/S1, bilateral iliac crests, and bilateral posterior superior iliac spines, in order to define the trunk and pelvis. Based on these markers, a total of 15 body segments were modeled, including the upper arms, forearms, hands, head, trunk, pelvis, thighs, shanks, and feet. 

Once instrumented, the subjects performed the pose sequences, while guided by their instructor. The sequence of the poses was the same as when it was carried out in the regular yoga classes. A firm but portable clear Plexiglas wall, which permitted the capture of the markers, was positioned for wall support in the lab visits. For each pose, the participant was instructed to begin in a starting position, move smoothly into the pose, hold the pose while taking one full breath, and then return back to the original position. Simultaneously, the instructor also performed each pose in order to provide visual cueing. Once the participant had moved into the pose position, the instructor provided a verbal cue to the research associate to initiate the 3-second data collection. Two successful trials of each pose version were collected, and all 3 seconds of each pose were used for the analyses. 

GRFs were measured from a force platform at 1560 Hz (AMTI, Watertown, MA). Qualisys Track Manager Software (Qualisys, Gothenburg, Sweden) and Visual 3D (C-motion, Rockville, MD) were used to process the raw coordinate data and compute segmental kinematics and kinetics. Trajectory data was filtered with a fourth-order zero lag Butterworth 12 Hz low-pass filter. In Visual 3D, the head was modeled as a sphere, the torso and pelvis as cylinders, and the upper and lower extremity segments as frusta of cones. The local coordinate systems of body segments were derived from the standing calibration trial. Joint kinematics were computed based upon Euler angles with the following order of rotations: flexion/extension, abduction/adduction, and internal/external rotation. The principle moments of inertia were determined from the subject's total body weight, segment geometry, and anthropometric data. Using standard inverse dynamics techniques, along with the International Society of Biomechanics recommended coordinate systems, net JMOFs in the sagittal and frontal planes, for the ankle, knee and hip, were calculated from the inertial properties, segmental kinematics, and GRFs. JMOFs were normalized to each subject's body weight in kg. 

Surface electromyographic (EMG) signals of the lower gluteus medius, hamstrings, vastus lateralis, gastrocnemius, rectus abdominis, and erector spinae muscles were collected on the subjects' dominant limb at 1560 Hz using active surface electrodes (Motion Lab Systems, Baton Rouge, LA). The electrodes were placed in the center of the muscle bellies with the electrodes aligned in the direction of the muscle fibers. The obtained EMG signals were amplified (×1000), notch filtered at 60 Hz, and band-pass filtered at 20–500 Hz. A root mean square smoothing algorithm with a 75 ms constant window was used to smooth the EMG data over the 3-second data collection period corresponding to the epoch of kinematic and kinetic data. EMG processing and smoothing were performed using MATLAB (MathWorks, Natick, MA). EMG readings quantify the muscle activation patterns associated with performance of the postures. In some cases, the EMG readings augment the JMOF results. For example, a high knee JMOF together with a high quadriceps EMG activation amplitude mutually support that the quadriceps are undergoing substantial loading as a result of the asana. In some instances, EMG provides unique information about muscle groups that are difficult to quantify with JMOFs, such as abdominal and spinal muscle groups. 

**Figure 1:**
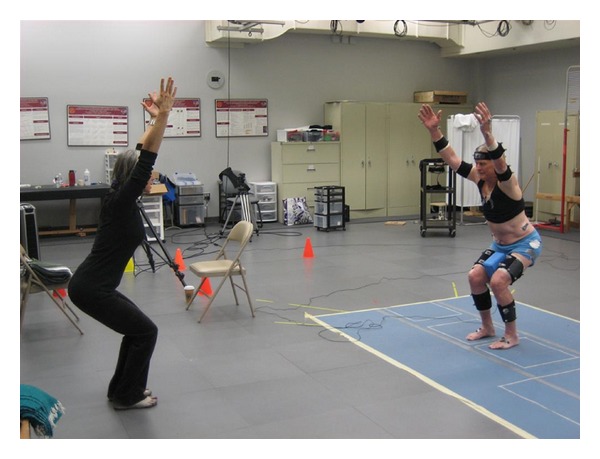
YESS participant performing the intermediate chair asana while instrumented for biomechanical analysis.

Once instrumented, the participants performed the asana sequences, guided by their instructor (Figure 1). Sequences were the same as those used during the regular yoga classes. A firm but portable clear Plexiglas wall was positioned for wall support in the lab visits. The clear wall permitted the capture of the markers. We decided *a priori* to examine the asanas while the participants were holding the poses in a static position; this provides information regarding the physical demands of the postures themselves. For each posture, the participant began in a starting position, moved smoothly into the posture, held the posture while taking one full breath, and then returned back to the original position. The instructor provided visual cues by demonstrating the postures simultaneously. Once the participant moved into the position, the instructor provided a verbal cue to the research associate to initiate the 3-second data collection. Two trials of each asana were collected. For postures that involved asymmetric positioning of the 2 support limbs (e.g., side stretch, crescent, and warrior asanas), the postures were done twice—initially with the dominant limb in the front (leading) position and subsequently in the back (trailing) position. The JMOFs varied considerably between the leading and trailing limbs; thus, they were considered separately. Consequently, side stretch, crescent, and warrior asanas were subdivided into leading- and trailing-limb postures (e.g., crescent front and crescent back). The participants also completed 2 walking trials at their self-selected speed, in order to provide a reference condition, that is, walking is a well-studied, stereotypical activity about which we have a lot of biomechanical data as well as a common intuitive understanding of demand. To provide a standardized frame of reference, the EMG measured during each posture was “normalized” to the EMG that resulted from walking, by dividing the maximum EMG signal developed during the posture on the maximum EMG signal invoked during walking.

## 3. Results

### 3.1. Biomechanical Profiles

Peak JMOF and maximum joint angle data were averaged across the 2 trials and the 20 participants of each posture. EMG data for each muscle group were normalized to the average peak signal generated during the walking trials and then averaged across the 2 posture trials and the 20 participants. The combined kinematic, kinetic, and EMG data were then organized into individual asana profiles, which also include a photograph of the posture and a figure of the skeletal model.

Biomechanical profiles of the 9 introductory and 12 intermediate asanas (including leading and trailing limbs) are presented in Figures 2–22. We provide here three illustrative examples of how to interpret these individual profiles. 

### 3.2. Targeting of the Knee Extensors

**Figure 2:**
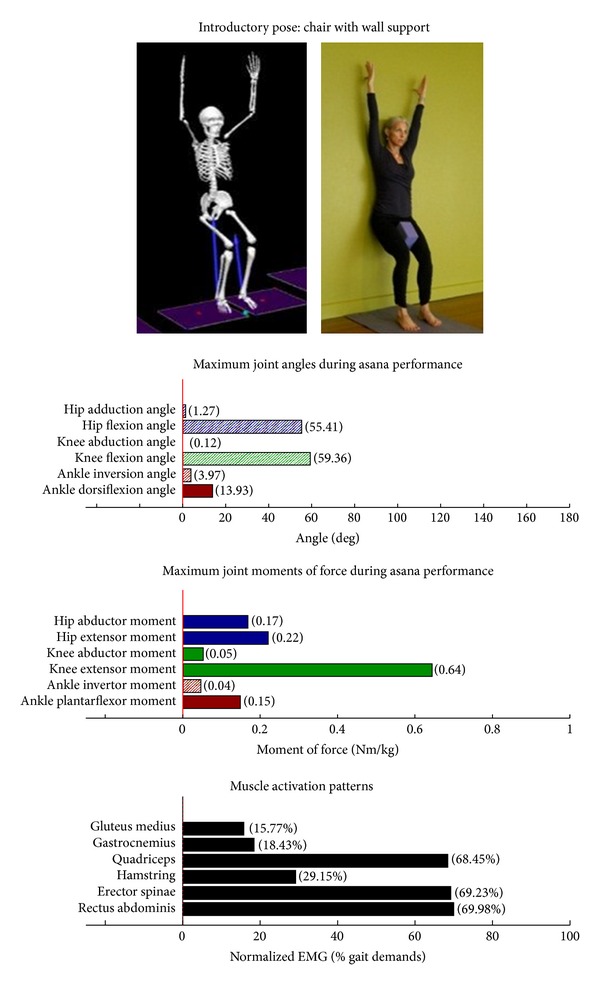
Asana physical demands. Biomechanical profiles: average maximum ankle, knee, and hip joint angles and joint moments of force (JMOFs) engendered during the middle 3 seconds of asana performance. Hashed bars represent hip adductor and flexor, knee adductor and flexor, and ankle invertor angles and JMOFs; whereas, the open bars represent hip abductor and extensor, knee abductor and extensor, and ankle evertor angles and JMFs. Muscle activation patterns represent the average peak EMG signals generated during the middle 3 seconds of asana performance. These signals were normalized to the peak EMG signals generated during each participant's walking trials at a self-selected pace.

Postures which are likely to stimulate adaptation of the knee extensor muscles (quadriceps) are those that developed appreciable *knee extensor* JMOFs and quadriceps EMG activity. These include the introductory postures chair with wall support, warrior I and II front limb (Figures 2, 5, and 7, resp.). Intermediate postures which targeted the knee extensors included chair, warrior II front, crescent front, and crescent back (Figures 11, 14, 21, and 22, resp.).

### 3.3. Targeting of the Hip Abductors

**Figure 3:**
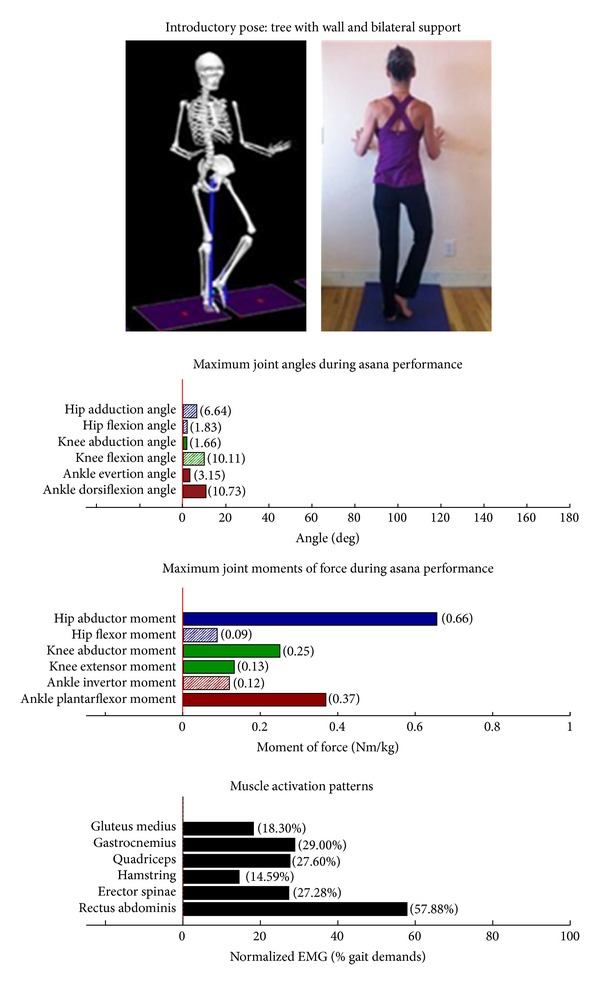
Asana physical demands. Biomechanical profiles: average maximum ankle, knee, and hip joint angles and joint moments of force (JMOFs) engendered during the middle 3 seconds of asana performance. Hashed bars represent hip adductor and flexor, knee adductor and flexor, and ankle invertor angles and JMOFs; whereas, the open bars represent hip abductor and extensor, knee abductor and extensor, and ankle evertor angles and JMFs. Muscle activation patterns represent the average peak EMG signals generated during the middle 3 seconds of asana performance. These signals were normalized to the peak EMG signals generated during each participant's walking trials at a self-selected pace.

**Figure 4:**
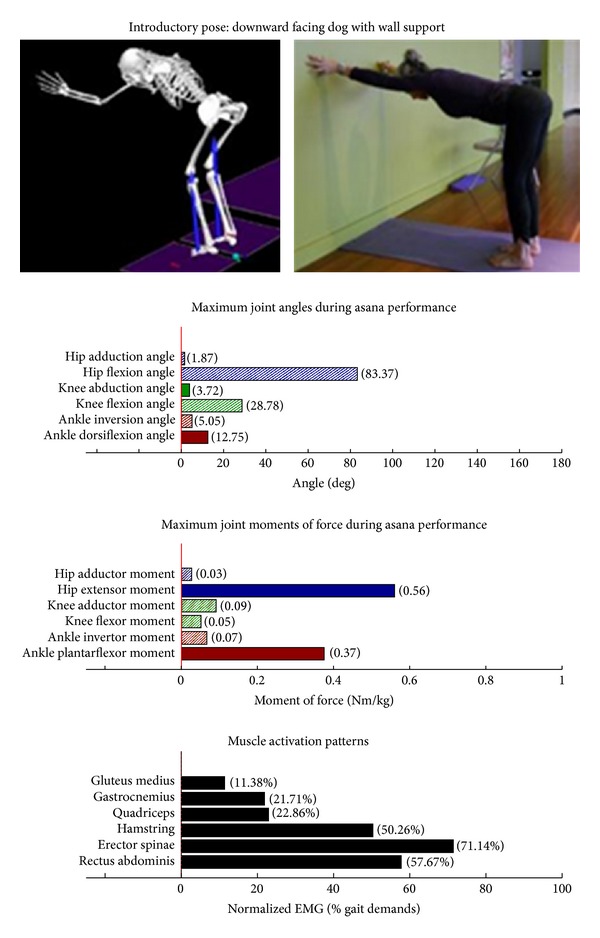
Asana physical demands. Biomechanical profiles: average maximum ankle, knee, and hip joint angles and joint moments of force (JMOFs) engendered during the middle 3 seconds of asana performance. Hashed bars represent hip adductor and flexor, knee adductor and flexor, and ankle invertor angles and JMOFs; whereas, the open bars represent hip abductor and extensor, knee abductor and extensor, and ankle evertor angles and JMFs. Muscle activation patterns represent the average peak EMG signals generated during the middle 3 seconds of asana performance. These signals were normalized to the peak EMG signals generated during each participant's walking trials at a self-selected pace.

**Figure 5:**
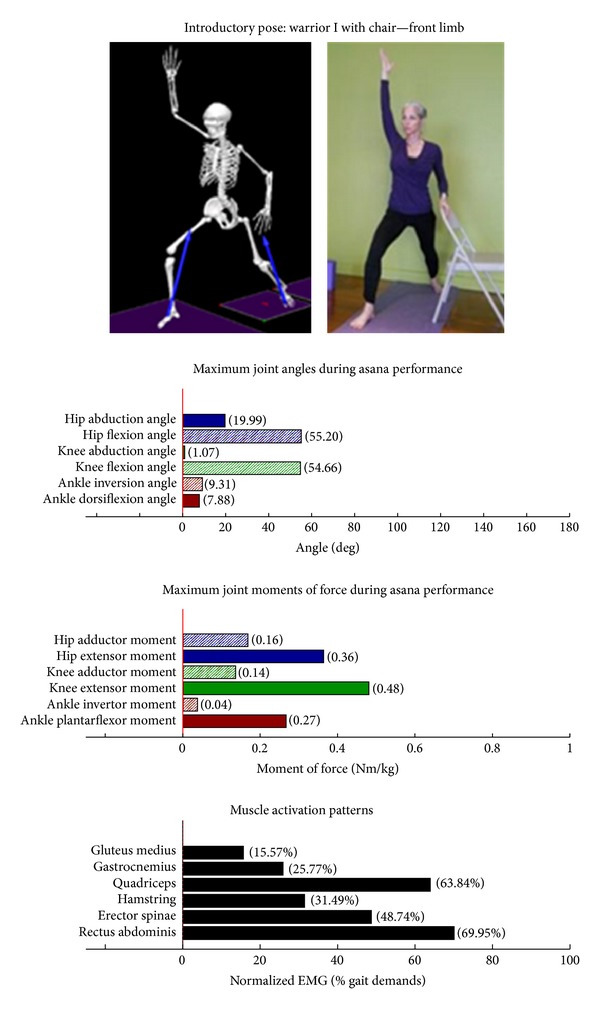
Asana physical demands. Biomechanical profiles: average maximum ankle, knee, and hip joint angles and joint moments of force (JMOFs) engendered during the middle 3 seconds of asana performance. Hashed bars represent hip adductor and flexor, knee adductor and flexor, and ankle invertor angles and JMOFs; whereas, the open bars represent hip abductor and extensor, knee abductor and extensor, and ankle evertor angles and JMFs. Muscle activation patterns represent the average peak EMG signals generated during the middle 3 seconds of asana performance. These signals were normalized to the peak EMG signals generated during each participant's walking trials at a self-selected pace.

**Figure 6:**
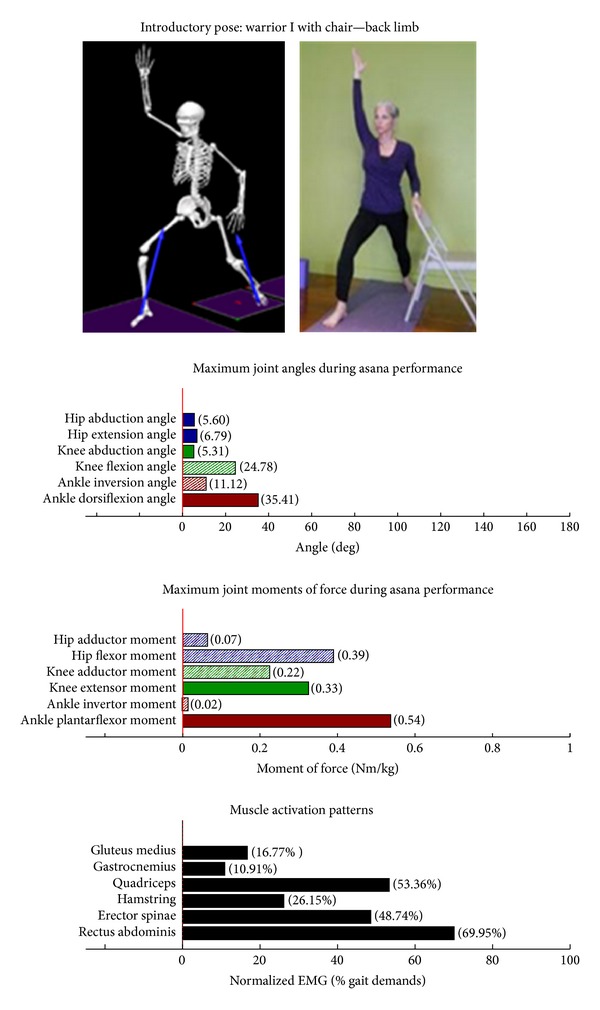
Asana physical demands. Biomechanical profiles: average maximum ankle, knee, and hip joint angles and joint moments of force (JMOFs) engendered during the middle 3 seconds of asana performance. Hashed bars represent hip adductor and flexor, knee adductor and flexor, and ankle invertor angles and JMOFs; whereas, the open bars represent hip abductor and extensor, knee abductor and extensor, and ankle evertor angles and JMFs. Muscle activation patterns represent the average peak EMG signals generated during the middle 3 seconds of asana performance. These signals were normalized to the peak EMG signals generated during each participant's walking trials at a self-selected pace.

**Figure 7:**
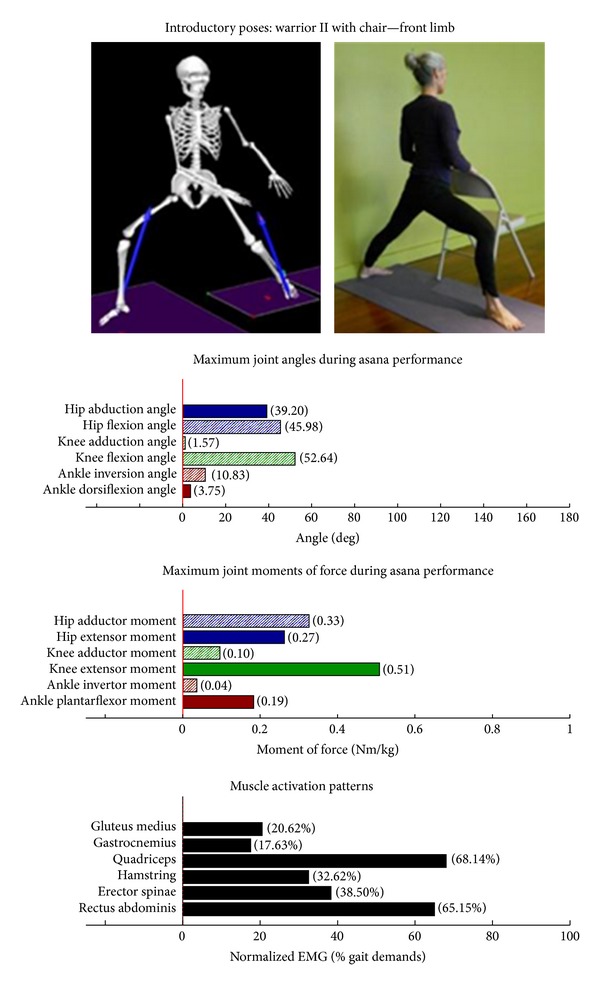
Asana physical demands. Biomechanical profiles: average maximum ankle, knee, and hip joint angles and joint moments of force (JMOFs) engendered during the middle 3 seconds of asana performance. Hashed bars represent hip adductor and flexor, knee adductor and flexor, and ankle invertor angles and JMOFs; whereas, the open bars represent hip abductor and extensor, knee abductor and extensor, and ankle evertor angles and JMFs. Muscle activation patterns represent the average peak EMG signals generated during the middle 3 seconds of asana performance. These signals were normalized to the peak EMG signals generated during each participant's walking trials at a self-selected pace.

**Figure 8:**
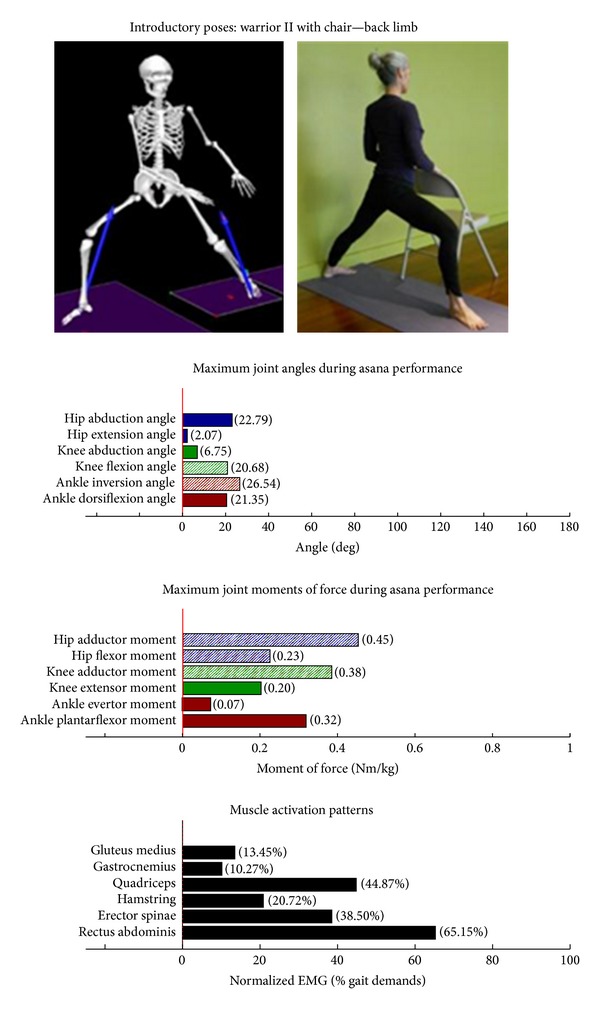
Asana physical demands. Biomechanical profiles: average maximum ankle, knee, and hip joint angles and joint moments of force (JMOFs) engendered during the middle 3 seconds of asana performance. Hashed bars represent hip adductor and flexor, knee adductor and flexor, and ankle invertor angles and JMOFs; whereas, the open bars represent hip abductor and extensor, knee abductor and extensor, and ankle evertor angles and JMFs. Muscle activation patterns represent the average peak EMG signals generated during the middle 3 seconds of asana performance. These signals were normalized to the peak EMG signals generated during each participant's walking trials at a self-selected pace.

**Figure 9:**
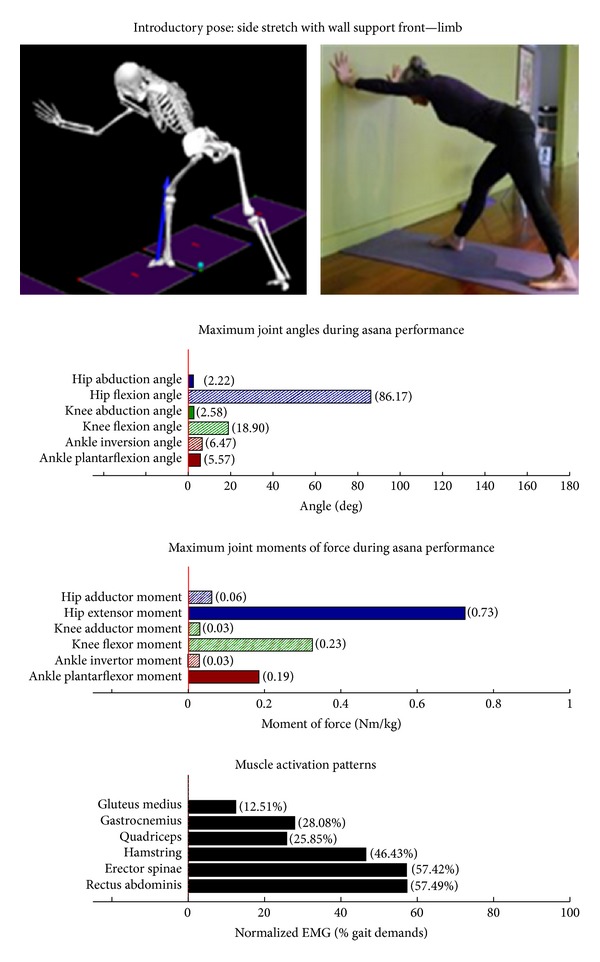
Asana physical demands. Biomechanical profiles: average maximum ankle, knee, and hip joint angles and joint moments of force (JMOFs) engendered during the middle 3 seconds of asana performance. Hashed bars represent hip adductor and flexor, knee adductor and flexor, and ankle invertor angles and JMOFs; whereas, the open bars represent hip abductor and extensor, knee abductor and extensor, and ankle evertor angles and JMFs. Muscle activation patterns represent the average peak EMG signals generated during the middle 3 seconds of asana performance. These signals were normalized to the peak EMG signals generated during each participant's walking trials at a self-selected pace.

**Figure 10:**
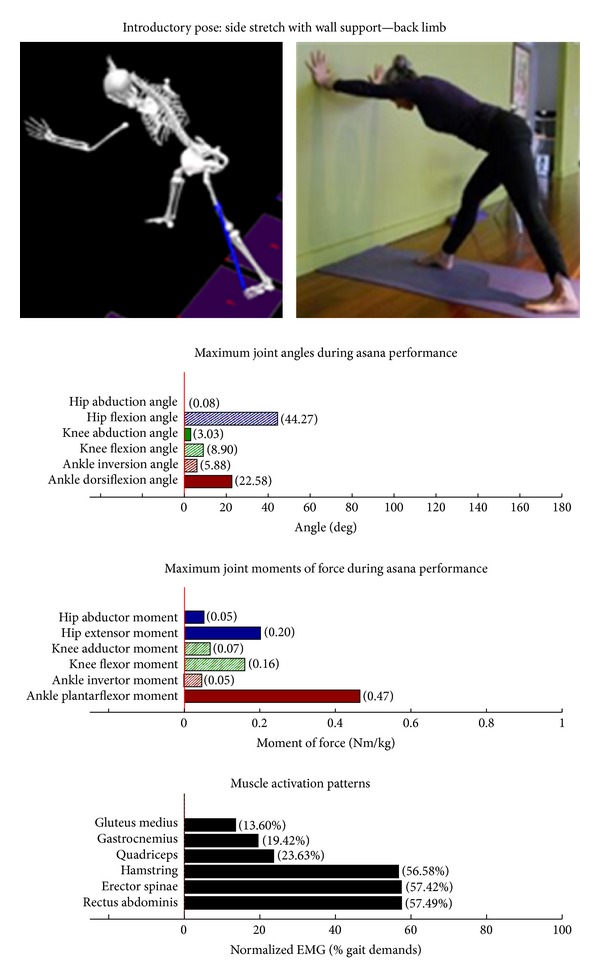
Asana physical demands. Biomechanical profiles: average maximum ankle, knee, and hip joint angles and joint moments of force (JMOFs) engendered during the middle 3 seconds of asana performance. Hashed bars represent hip adductor and flexor, knee adductor and flexor, and ankle invertor angles and JMOFs; whereas, the open bars represent hip abductor and extensor, knee abductor and extensor, and ankle evertor angles and JMFs. Muscle activation patterns represent the average peak EMG signals generated during the middle 3 seconds of asana performance. These signals were normalized to the peak EMG signals generated during each participant's walking trials at a self-selected pace.

**Figure 11:**
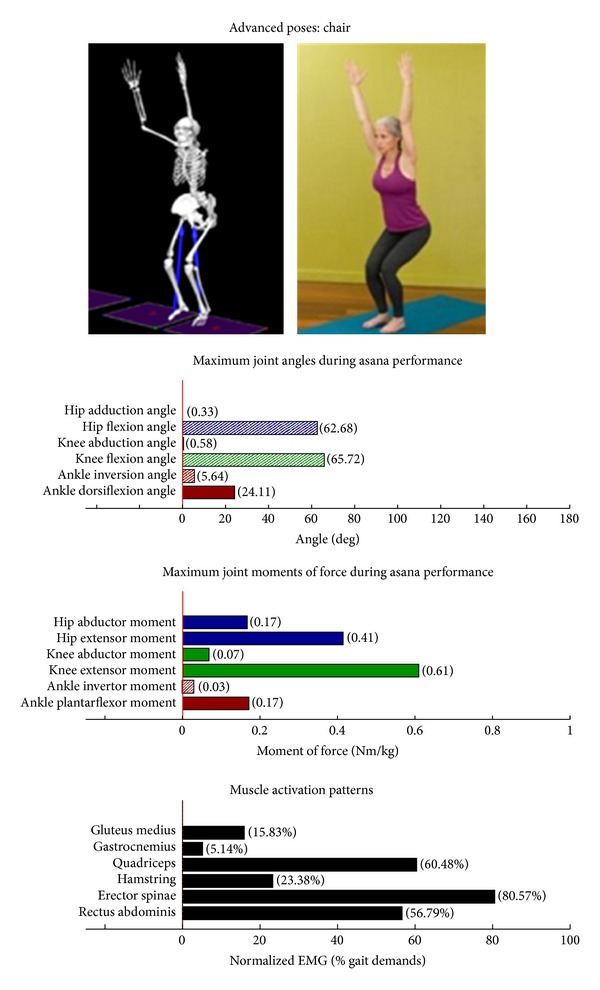
Asana physical demands. Biomechanical profiles: average maximum ankle, knee, and hip joint angles and joint moments of force (JMOFs) engendered during the middle 3 seconds of asana performance. Hashed bars represent hip adductor and flexor, knee adductor and flexor, and ankle invertor angles and JMOFs; whereas, the open bars represent hip abductor and extensor, knee abductor and extensor, and ankle evertor angles and JMFs. Muscle activation patterns represent the average peak EMG signals generated during the middle 3 seconds of asana performance. These signals were normalized to the peak EMG signals generated during each participant's walking trials at a self-selected pace.

**Figure 12:**
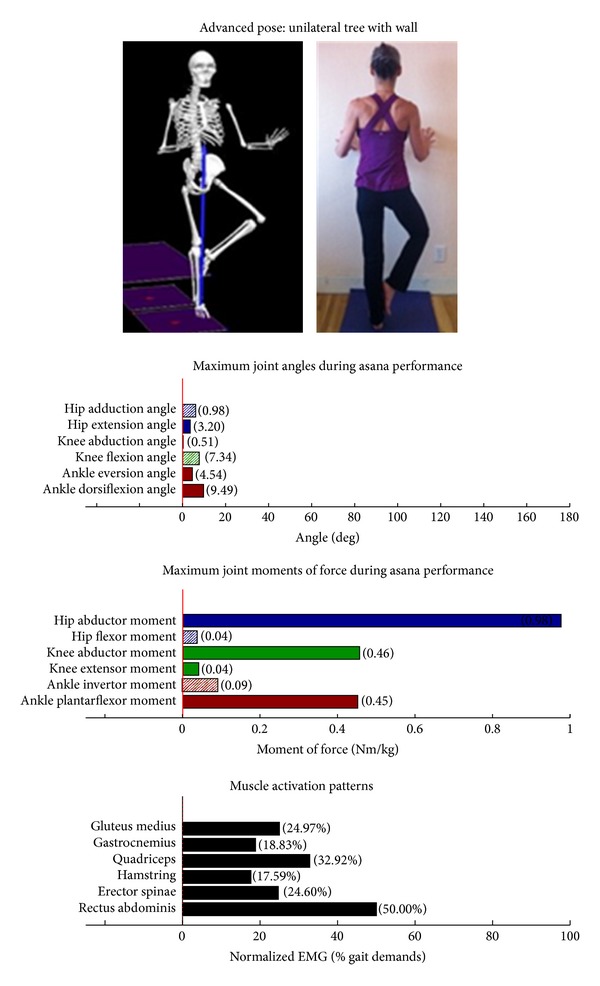
Asana physical demands. Biomechanical profiles: average maximum ankle, knee, and hip joint angles and joint moments of force (JMOFs) engendered during the middle 3 seconds of asana performance. Hashed bars represent hip adductor and flexor, knee adductor and flexor, and ankle invertor angles and JMOFs; whereas, the open bars represent hip abductor and extensor, knee abductor and extensor, and ankle evertor angles and JMFs. Muscle activation patterns represent the average peak EMG signals generated during the middle 3 seconds of asana performance. These signals were normalized to the peak EMG signals generated during each participant's walking trials at a self-selected pace.

**Figure 13:**
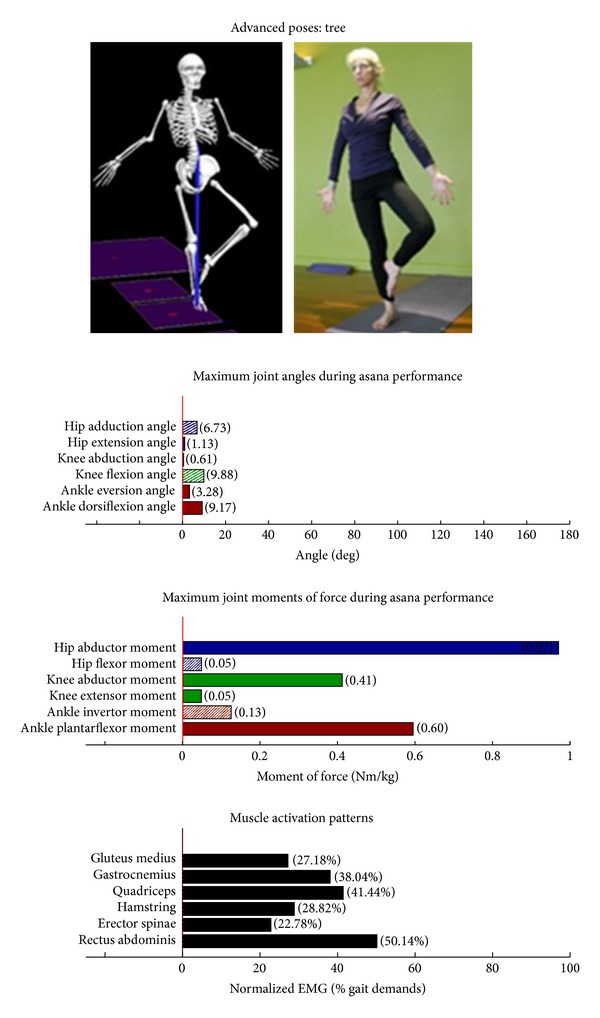
Asana physical demands. Biomechanical profiles: average maximum ankle, knee, and hip joint angles and joint moments of force (JMOFs) engendered during the middle 3 seconds of asana performance. Hashed bars represent hip adductor and flexor, knee adductor and flexor, and ankle invertor angles and JMOFs; whereas, the open bars represent hip abductor and extensor, knee abductor and extensor, and ankle evertor angles and JMFs. Muscle activation patterns represent the average peak EMG signals generated during the middle 3 seconds of asana performance. These signals were normalized to the peak EMG signals generated during each participant's walking trials at a self-selected pace.

**Figure 14:**
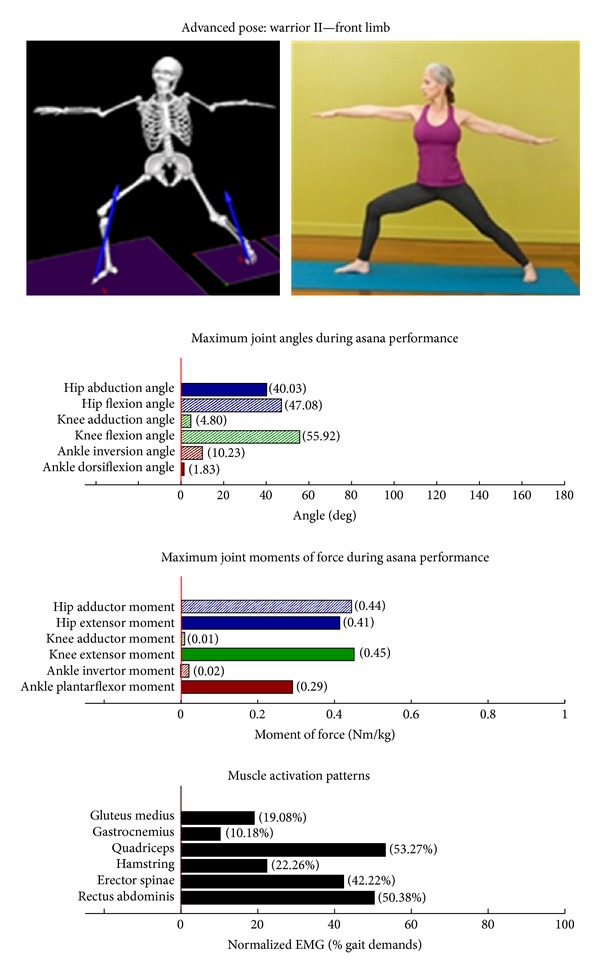
Asana physical demands. Biomechanical profiles: average maximum ankle, knee, and hip joint angles and joint moments of force (JMOFs) engendered during the middle 3 seconds of asana performance. Hashed bars represent hip adductor and flexor, knee adductor and flexor, and ankle invertor angles and JMOFs; whereas, the open bars represent hip abductor and extensor, knee abductor and extensor, and ankle evertor angles and JMFs. Muscle activation patterns represent the average peak EMG signals generated during the middle 3 seconds of asana performance. These signals were normalized to the peak EMG signals generated during each participant's walking trials at a self-selected pace.

**Figure 15:**
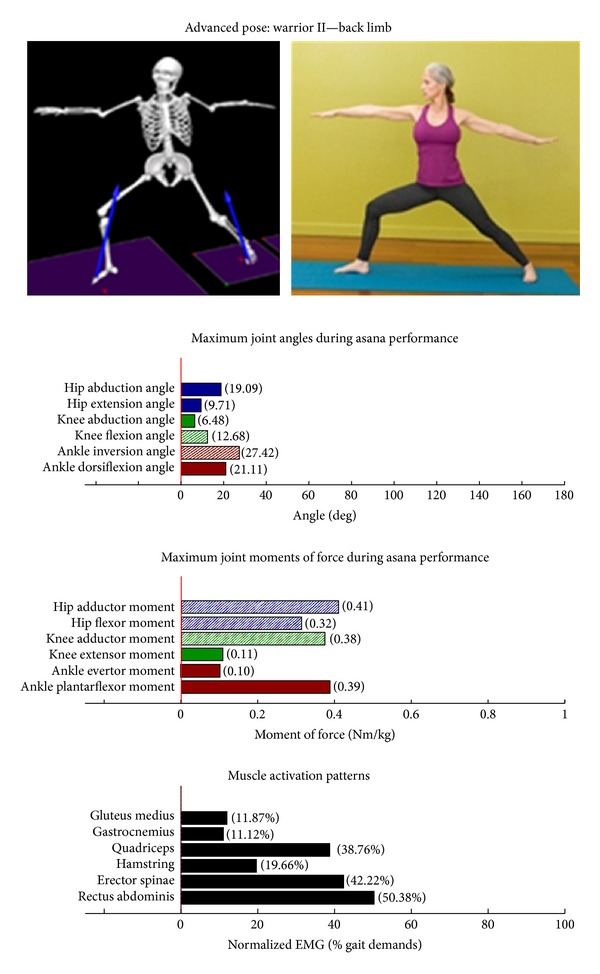
Asana physical demands. Biomechanical profiles: average maximum ankle, knee, and hip joint angles and joint moments of force (JMOFs) engendered during the middle 3 seconds of asana performance. Hashed bars represent hip adductor and flexor, knee adductor and flexor, and ankle invertor angles and JMOFs; whereas, the open bars represent hip abductor and extensor, knee abductor and extensor, and ankle evertor angles and JMFs. Muscle activation patterns represent the average peak EMG signals generated during the middle 3 seconds of asana performance. These signals were normalized to the peak EMG signals generated during each participant's walking trials at a self-selected pace.

**Figure 16:**
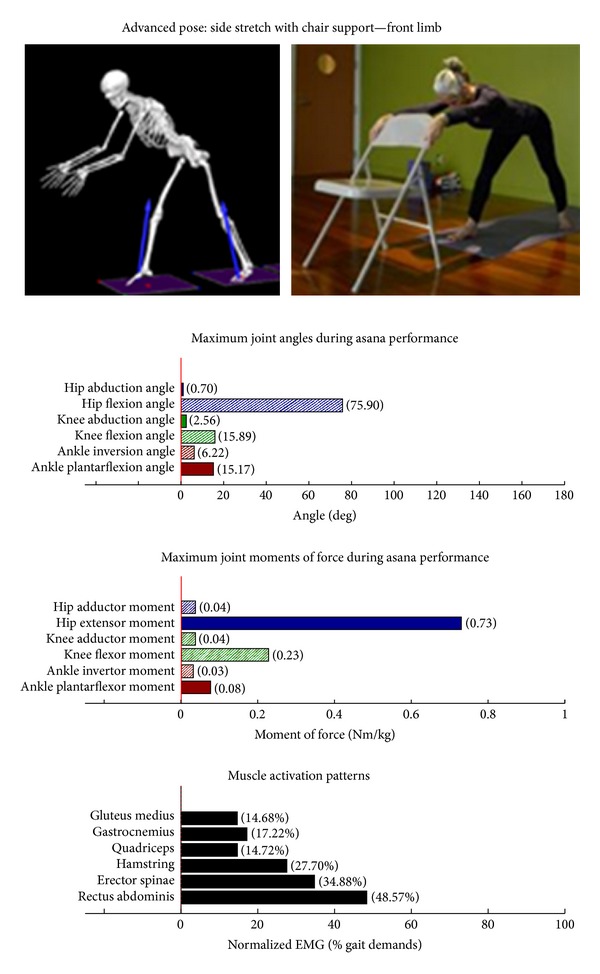
Asana physical demands. Biomechanical profiles: average maximum ankle, knee, and hip joint angles and joint moments of force (JMOFs) engendered during the middle 3 seconds of asana performance. Hashed bars represent hip adductor and flexor, knee adductor and flexor, and ankle invertor angles and JMOFs; whereas, the open bars represent hip abductor and extensor, knee abductor and extensor, and ankle evertor angles and JMFs. Muscle activation patterns represent the average peak EMG signals generated during the middle 3 seconds of asana performance. These signals were normalized to the peak EMG signals generated during each participant's walking trials at a self-selected pace.

**Figure 17:**
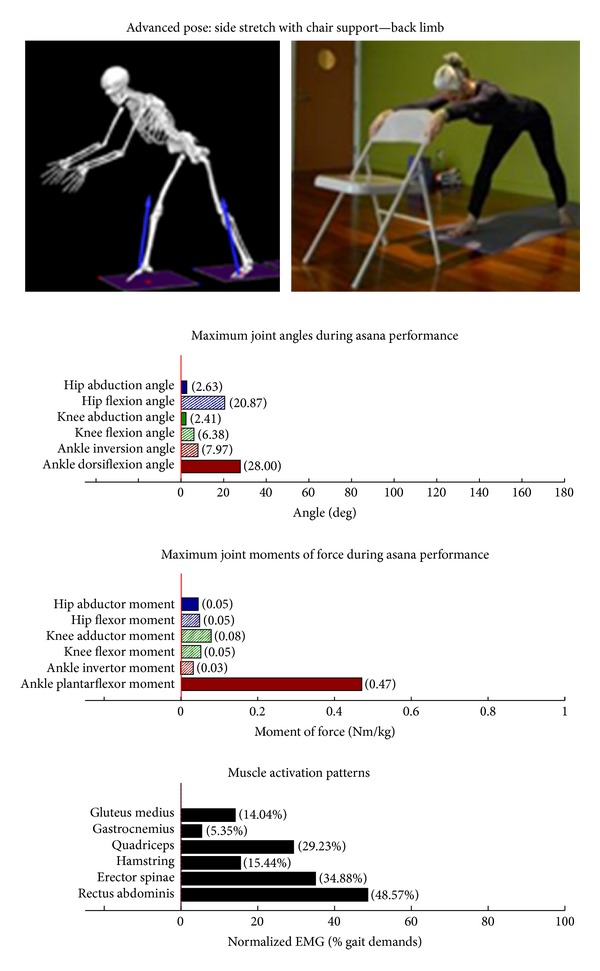
Asana physical demands. Biomechanical profiles: average maximum ankle, knee, and hip joint angles and joint moments of force (JMOFs) engendered during the middle 3 seconds of asana performance. Hashed bars represent hip adductor and flexor, knee adductor and flexor, and ankle invertor angles and JMOFs; whereas, the open bars represent hip abductor and extensor, knee abductor and extensor, and ankle evertor angles and JMFs. Muscle activation patterns represent the average peak EMG signals generated during the middle 3 seconds of asana performance. These signals were normalized to the peak EMG signals generated during each participant's walking trials at a self-selected pace.

**Figure 18:**
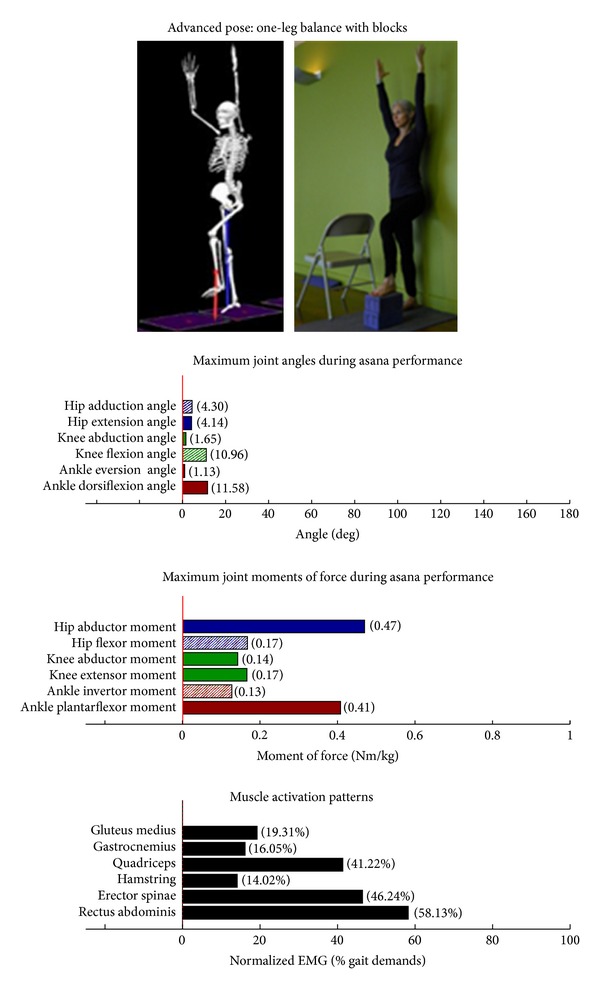
Asana physical demands. Biomechanical profiles: average maximum ankle, knee, and hip joint angles and joint moments of force (JMOFs) engendered during the middle 3 seconds of asana performance. Hashed bars represent hip adductor and flexor, knee adductor and flexor, and ankle invertor angles and JMOFs; whereas, the open bars represent hip abductor and extensor, knee abductor and extensor, and ankle evertor angles and JMFs. Muscle activation patterns represent the average peak EMG signals generated during the middle 3 seconds of asana performance. These signals were normalized to the peak EMG signals generated during each participant's walking trials at a self-selected pace.

**Figure 19:**
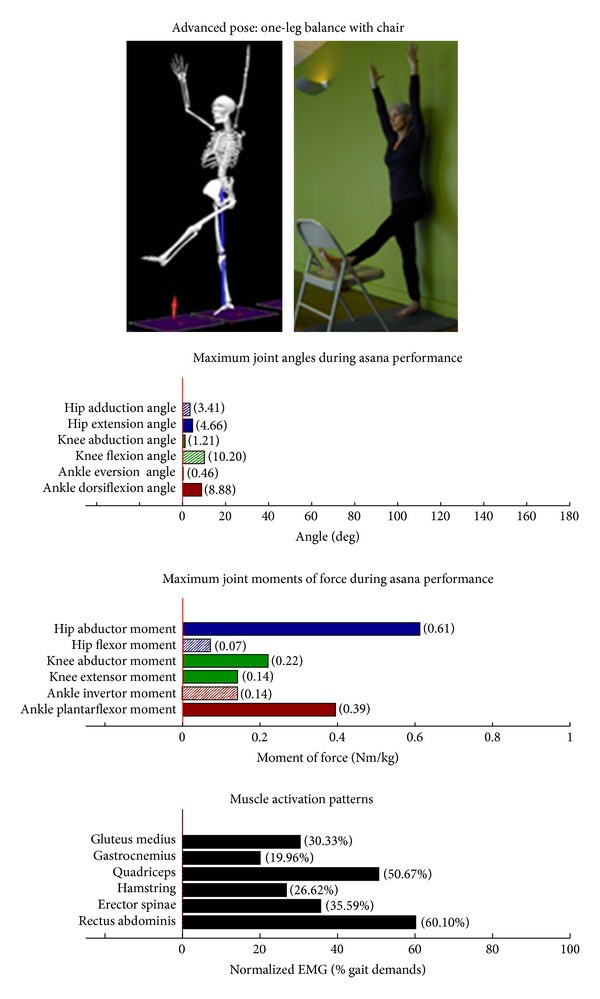
Asana physical demands. Biomechanical profiles: average maximum ankle, knee, and hip joint angles and joint moments of force (JMOFs) engendered during the middle 3 seconds of asana performance. Hashed bars represent hip adductor and flexor, knee adductor and flexor, and ankle invertor angles and JMOFs; whereas, the open bars represent hip abductor and extensor, knee abductor and extensor, and ankle evertor angles and JMFs. Muscle activation patterns represent the average peak EMG signals generated during the middle 3 seconds of asana performance. These signals were normalized to the peak EMG signals generated during each participant's walking trials at a self-selected pace.

**Figure 20:**
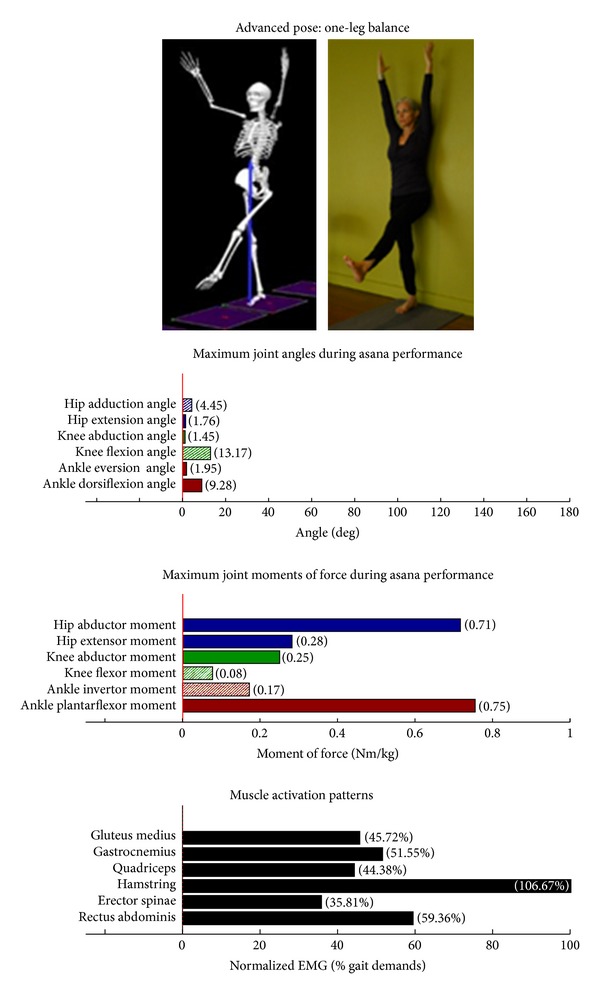
Asana physical demands. Biomechanical profiles: average maximum ankle, knee, and hip joint angles and joint moments of force (JMOFs) engendered during the middle 3 seconds of asana performance. Hashed bars represent hip adductor and flexor, knee adductor and flexor, and ankle invertor angles and JMOFs; whereas, the open bars represent hip abductor and extensor, knee abductor and extensor, and ankle evertor angles and JMFs. Muscle activation patterns represent the average peak EMG signals generated during the middle 3 seconds of asana performance. These signals were normalized to the peak EMG signals generated during each participant's walking trials at a self-selected pace.

**Figure 21:**
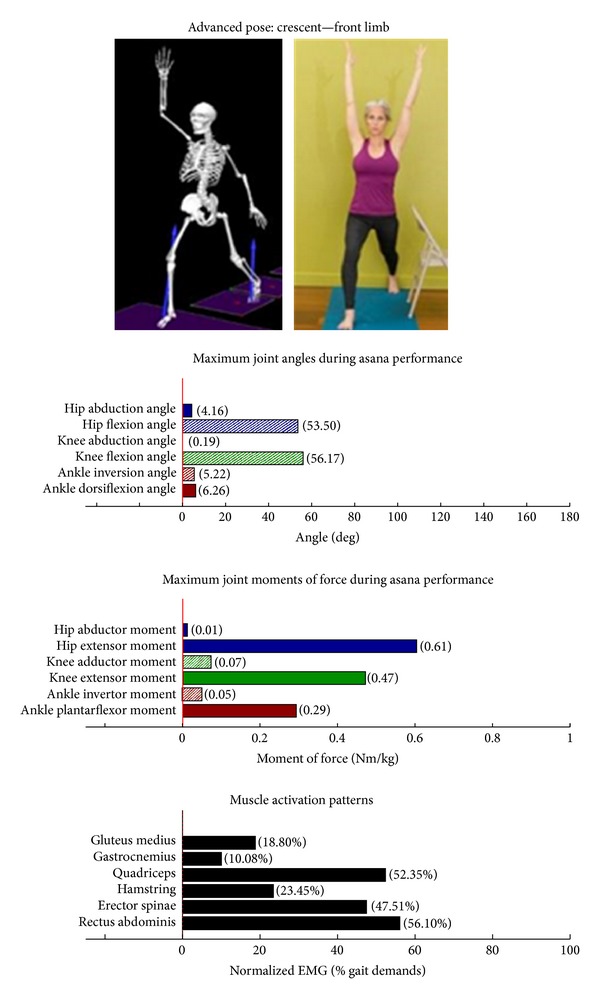
Asana physical demands. Biomechanical profiles: average maximum ankle, knee, and hip joint angles and joint moments of force (JMOFs) engendered during the middle 3 seconds of asana performance. Hashed bars represent hip adductor and flexor, knee adductor and flexor, and ankle invertor angles and JMOFs; whereas, the open bars represent hip abductor and extensor, knee abductor and extensor, and ankle evertor angles and JMFs. Muscle activation patterns represent the average peak EMG signals generated during the middle 3 seconds of asana performance. These signals were normalized to the peak EMG signals generated during each participant's walking trials at a self-selected pace.

**Figure 22:**
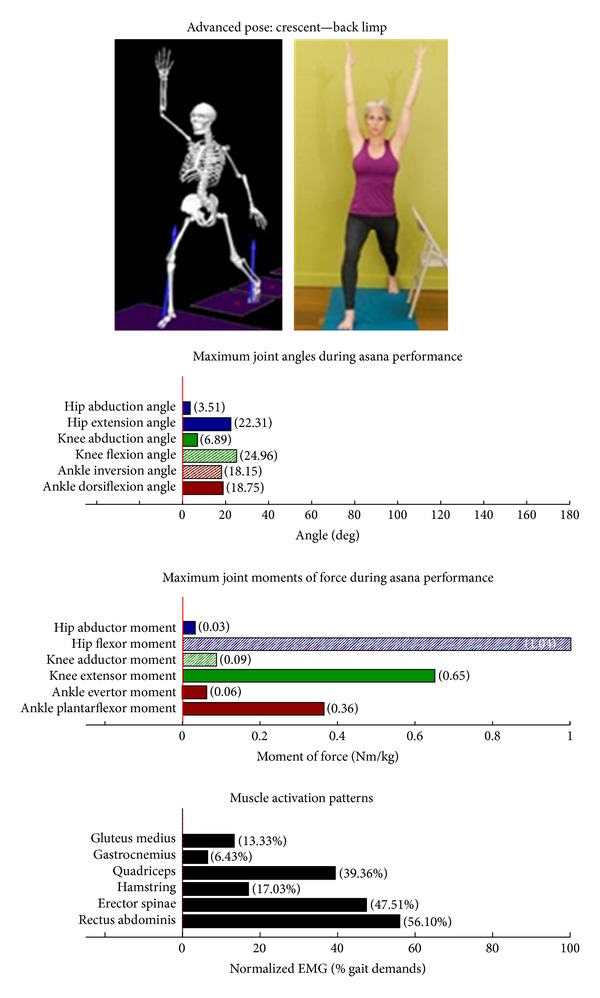
Asana physical demands. Biomechanical profiles: average maximum ankle, knee, and hip joint angles and joint moments of force (JMOFs) engendered during the middle 3 seconds of asana performance. Hashed bars represent hip adductor and flexor, knee adductor and flexor, and ankle invertor angles and JMOFs; whereas, the open bars represent hip abductor and extensor, knee abductor and extensor, and ankle evertor angles and JMFs. Muscle activation patterns represent the average peak EMG signals generated during the middle 3 seconds of asana performance. These signals were normalized to the peak EMG signals generated during each participant's walking trials at a self-selected pace.

Asanas which are likely to stimulate adaptation of the hip abductor muscles (gluteus medius, minimus, and superior gluteus maximus) are those postures that developed appreciable *hip abductor* JMOFs and gluteus medius EMG activity. Only one introductory posture, tree with bilateral and wall support (Figure 3), generated an appreciable hip abductor moment. Among the intermediate postures, tree with unilateral and wall support, tree, and all three one-leg balance postures, (Figures 12, 13, and 18–20, resp.) generated appreciable hip abductor JMOFs and gluteus medius EMG activity. Whereas there was little difference in the hip abductor JMOF between the two advanced tree postures, there was a progressive increase in the hip abductor JMOF and gluteus medius EMG activity generated across the three single-leg balance postures.

### 3.4. Targeting the Ankle Dorsiflexors

None of the 21 asanas examined (neither introductory nor intermediate) generated a dorsiflexor JMOF; rather all generated plantar-flexor JMOFs. Therefore, none of these postures are likely to stimulate adaptation of the ankle dorsiflexors (tibialis anterior, extensor digitorum, and extensor hallucis).

### 3.5. Targeting the Core Muscles: Erector Spinae and Rectus Abdominis

All of the postures examined (both introductory and intermediate) generated appreciable rectus abdominis EMG activity. Those postures generating appreciable erector spinae activity included the introductory chair with wall support, downward facing dog with wall support, warrior I with chair Support, warrior II with chair support, and side stretch with wall support (Figures 2 and 4–10). The intermediate postures chair, warrior II, side stretch with chair support, all 3 one-leg balance postures, and crescent, also induced appreciable erector spinae activity (Figures 11 and 14–22, resp.).

## 4. Discussion

This is the first study to use biomechanical investigation to quantify the physical demands of introductory- and intermediate-level Hatha asanas performed by seniors. Biomechanical profiles generated from this investigation can be used to inform experienced instructors in their design of yoga programs for older adults. Using this information, instructors and therapists, whom have specialized in training with senior populations, can select appropriate postures in order to create comprehensive programs which affect a variety of joints and muscle groups. They can also use this information to create balanced programs that prevent excessive repetitive loading of the same tissues and joint structures. Lastly, the profiles can be used to make evidenced-based decisions in order to specifically target weak muscle groups and/or avoid the loading of pathological articular and myotendinous tissues. For example, a yoga program that comprehensively addresses the functionally important muscle groups of the lower extremity needs to include postures that induce appreciable JMOFs across the hip, knee, and ankle, in multiple directions. Just as important, the program should not put participants at risk by repetitively loading the same muscular, tendinous, or articular tissues without providing appropriate recovery intervals.

Although our goal was to develop a “balanced” and comprehensive yoga program that targeted all of the functionally important muscle groups of the LE, the introductory program, in particular, was deficient in several areas. Most strikingly, none of the asanas developed an ankle dorsiflexor JMOF. The ankle dorsiflexors are important muscles which “lift the front of the foot” during the swing phase of gait in order to clear the toes and prevent tripping accidents. Not surprisingly, poor ankle dorsiflexor strength is associated with increased fall risk in community-dwelling older adults [14]. Our findings suggest that additional unstudied postures need to be biomechanically examined in order to identify those which develop ankle dorsiflexor JMOFs. Once identified, these should then be incorporated into senior programs. Else, additional nonyoga activities/exercises should be integrated into yoga programs in order to address dorsiflexor targeting.

Similarly, the introductory poses generated only modest hip abductor JMOFs. The hip abductors are important stabilizers of the pelvis, and their muscular performance is correlated with balance and fall risk in seniors [15–17]. Thus, we believe it would be prudent to identify additional “introductory level” postures, which target the hip abductors. In contrast to the introductory program, several intermediate asanas, including the tree with unilateral and wall support, tree without support, and all three one-leg balance postures, generated appreciable hip abductor JMOFs and gluteus medius activity. Notably, these were all single limb, standing postures. Few postures also targeted the hip flexors (warrior I, warrior II, and crescent, back limbs). The hip flexors are important in “pulling the limb forward” during the swing phase of gait and their performance is related to walking speed and fall recovery in older adults [18, 19]. 

Some of our findings were intuitive; for example, we demonstrated a progressive increase in the hip abductor JMOFs and gluteus medius EMG activity across the three one-leg balance postures. In other words, the JMOFs and gluteus medius EMG activity associated with the one-leg balance postures were the least with the block support, greater with the chair support, and greatest when the posture was done without additional support. On the other hand, some findings were counterintuitive; for example, we expected to see a similar progression in demand among the three tree postures. We found, however, that although there was a large difference in hip abductor JMOF and gluteus medius EMG activity between the introductory posture (tree with bilateral and wall support) and the intermediate posture (tree with unilateral and wall support), there was no difference between the tree with unilateral and wall support and the unsupported tree. This finding has important clinical implications; it suggests that the beginning participants can target their hip abductor muscles by lifting their contralateral foot off the floor and assisting their balance with use of a wall, even if they do not have the balance capabilities to hold the tree posture without the use of a wall. It also suggests that having participants let go of the wall, while potentially increasing their balance capabilities, is not likely to increase gluteus medius recruitment or performance.

Another interesting and unexpected finding was that all of the postures elicited appreciable rectus abdominis activity, which was up to 70% of that induced during walking—an activity requiring continuous dynamic control of the trunk. Contrastingly, erector spinae activity was more variable across the postures. Core stability is important because it influences trunk orientation which in turn affects hip, knee, and ankles position during yoga practice and joint kinematics during ambulation. Abdominal and erector strengthening exercises improve spinal mobility, balance, and functional mobility in seniors [20].

The profiles can also be used to identify those asanas, which might put seniors at risk for injury or exacerbate existing arthritic conditions. For example, frontal-plane JMOFs at the knee joint will increase the compressional forces across the tibia and femur and may exacerbate knee OA. The warrior postures, in particular, generated relatively large adductor JMOFs which are likely to increase loading on the *lateral* meniscus and tibiofemoral condyles, as well as the medial collateral ligament. Similarly, the tree postures also generated appreciable frontal-plane JMOFs at the knee; however, these were abductor JMOF's and thus likely to increase the loading across the *medial* meniscus and tibiofemoral condyles, and lateral collateral ligament.

Finally, the posture profiles may be used to generate appropriate sequences, so that the same musculoskeletal tissues are not loaded continuously without proper rest. For example, the chair and warrior front postures, both generated relatively large knee extensor JMOFs and quadriceps EMG activity; thus, it would be prudent not to sequence these postures successively but rather to sequence another posture in between these two, the side stretch posture, for example, which generated a knee *flexor* JMOF, would be a good candidate.

Among the limitations of this study is the limited number of asanas and modifications that we examined. The Hatha yoga type, in particular, lends itself well to the use of modified postures and participant-specific program development because there is not a *standard series* associated with Hatha. Thus, there are virtually hundreds of postures and modifications that can be used in senior programs, and future investigations should examine additional postures and modifications which are commonly used.

We also recognize that yoga is much more than just a series of postures but also incorporates breathing, meditation, chanting, and/or spiritual components. Although we attempted to remain as “true to the discipline” as possible, and therefore included opening and closing sequences, controlled breathing during the asanas, and the use of an instructor in the laboratory, we were only able to quantify the physical demands of the asanas and did not attempt to characterize these other important attributes.

Lastly, we focused our study on the physical demands associated with the postures during the 3-second interval the posture was held. We did not examine the physical demands associated with the transitions between the postures. In a recent pilot study conducted in our laboratory (unpublished data) we found that the JMOFs can be much greater during the transition from one posture to another, than during performance of the actual posture itself. Therefore, future studies should also examine asana transitions in order to provide additional sequencing information.

In conclusion, our findings demonstrate that introductory and advanced Hatha yoga postures engender a range of appreciable joint angles, JMOFs, and muscle activities about the ankle, knee, and hip. Further, we demonstrated that although our goal was to develop a “balanced” and comprehensive yoga program, the program was deficient in several areas. For example, none of the asanas developed an ankle dorsiflexor JMOF, and few developed hip flexor JMOFs. We also demonstrated that some findings were counterintuitive; for example, there was not a difference between the JMOF engendered during the tree with unilateral and wall support and the unsupported tree. Lastly, we found that all of the poses elicited appreciable rectus abdominis activity.

Profiles generated from this information may be used by experienced instructors and therapists, whom have specialized in training with senior populations, to appropriately design yoga programs for seniors that are comprehensive, safe, target specific muscle groups, unload articular structures at risk, and prevent repetitive overloading of musculoskeletal tissues. Additional randomized and controlled trial studies are needed to determine if evidenced-based programs, designed using biomechanical data from profiles like these, reduce adverse events and improve participant health outcomes. Similar biomechanical studies should also be designed to examine additional yoga postures, other yoga types (e.g., Raja), additional styles (e.g., Bikram), and the transitions between postures.
